# Multivariate data validation for investigating primary HCMV infection in pregnancy

**DOI:** 10.1016/j.dib.2016.08.050

**Published:** 2016-08-31

**Authors:** Luigi Barberini, Antonio Noto, Luca Saba, Francesco Palmas, Vassilios Fanos, Angelica Dessì, Maurizio Zavattoni, Claudia Fattuoni, Michele Mussap

**Affiliations:** a Department of Public Health Clinical and Molecular Medicine, University of Cagliari, Cagliari I-09042, Italy; b Department of Surgical Sciences, University of Cagliari and Neonatal Intensive Care Unit, Puericulture Institute and Neonatal Section, Azienda Ospedaliera Universitaria, Cagliari I-09042, Italy; c Department of Radiology, AOU University-Hospital, Cagliari I-09042, Italy; d Department of Chemical and Geological Sciences, University of Cagliari, I-09042 Italy; e Molecular Virology Unit, Microbiology and Virology Department, Fondazione Istituto di Ricovero e Cura a Carattere Scientifico (IRCCS) Policlinico San Matteo, Pavia, Italy; f Laboratory Medicine Service, IRCCS AOU San Martino-IST, University-Hospital, National Institute for Cancer Research, Genoa, Italy

**Keywords:** Metabolomics, Cytomegalovirus, Amniotic fluid, Pregnancy, Multivariate statistical approach, Cross validation performance, Partial, least square discriminant (PLS-DA) analysis

## Abstract

We reported data concerning the Gas Chromatography–Mass Spectrometry (GC–MS) based metabolomic analysis of amniotic fluid (AF) samples obtained from pregnant women infected with Human Cytomegalovirus (HCMV). These data support the publication “Primary HCMV Infection in Pregnancy from Classic Data towards Metabolomics: an Exploratory analysis” (C. Fattuoni, F. Palmas, A. Noto, L. Barberini, M. Mussap, et al., 2016) [2]. GC–MS and Multivariate analysis allow to recognize the molecular phenotype of HCMV infected fetuses (transmitters) and that of HCMV non-infected fetuses (non-transmitters); moreover, GC–MS and multivariate analysis allow to distinguish and to compare the molecular phenotype of these two groups with a control group consisting of AF samples obtained in HCMV non-infected pregnant women. The obtained data discriminate controls from transmitters as well as from non-transmitters; no statistically significant difference was found between transmitters and non-transmitters.

**Specifications Table**

**Table:** 

Subject area	*Metabolomics*
More specific subject area	*Clinical Metabolomics*
Type of data	*GC–MS data matrix, Figures, Tables*
How data was acquired	*Agilent 5975C mass spectrometer interfaced to the 7820 gas chromatograph. MetaboAnalyst web-tool*
Data format	*Processed*
Experimental factors	*After amniocentesis, AF samples were stored at 4 °C for 1-3 h maximum, aliquoted as a whole (without centrifugation) and transferred at −80 °C.*
Experimental features	*Samples were derivatized and analyzed by GC–MS. Metabolites were identified by AMDIS using GMD and in-house made libraries. Multivariate analysis was performed by MetaboAnalyst 3.0.*
Data source location	*Pavia, Italy*
Data accessibility	*Data are within this article*

**Value of the data**

• GC–MS analysis enabled to detect 58 metabolites; 50 of them have been accurately identified. To our knowledge, our data matrix is the first report on AF characterization in maternal HCMV infection.
• These data open new insights on the clinical utilization of the AF sample as diagnostic biofluid for metabolomics investigations.
• We applied the Receiving Operating Characteristic (ROC) analysis, based on the cross validation (CV) strategy. CV provides an unbiased assessment of the model without reducing the training data set; in other words, CV avoids overfitting because the training sample is independent from the validation sample. Ultimately, CV selects the algorithm with the smallest estimated risk. All these data can be useful for further design of experiments on this topic.

## 1 Data

The GC–MS data analysis of AF samples originated a matrix spreadsheet (Microsoft Excel^®^, Microsoft Co, Redmond, WA, USA) containing the detected metabolites and their respective concentrations (Supplementary Data). Columns contain: human metabolome database identification (HMDB-ID) [1], metabolites name and, from #1 to #64, sample identification. Data in black correspond to healthy controls (*n*=23); those in red to transmitters (*n*=20); and those in blue to non-transmitters (*n*=21).

## 2 Experimental design, materials and methods

Data originated from the analysis of AF samples obtained from HCMV infected women. The study population included pregnant women transmitting the infection to the fetus and pregnant women not transmitting the infection. In the transmitters population two subgroups, symptomatic and asymptomatic, have been examined. All AF samples were obtained after amniocentesis at the Departments of Obstetrics and Gynecology, Fondazione Istituto di Ricovero e Cura a Carattere Scientifico (IRCCS) Policlinico San Matteo, Pavia, Italy and were analyzed as reported previously [2]. The multivariate models were built by using the Partial Least Square-Discriminant Analysis (PLS-DA) (MetaboAnalyst 3.0, http://www.metaboanalyst.ca/) [3], [4], [5], as detailed in the research paper [2]. Power Analysis test, notably permutation test and the optimal sample size, was applied to assess the needful sample size for detecting the effect of interest with a given degree of confidence.

### 2.1 Data analysis and validation

The overall selected mothers were 63; however, one transmitter mother had a twin pregnancy: the first one baby was HCMV-infected (sample #46) and the second one was not (sample #61). Metabolites termed U483, U1437, U1751 and U1804 are unknown; they were found in most samples. Metabolites termed A192013, A196015, A203003, and A203005 have been defined by the MPIMP ID in the Golm Metabolome Database (GMD). By using the “Spectrum Library Search & Prediction of Functional Groups” web-tool [6], we found that these metabolites matched with unknown metabolites stored in GMD. All the remaining metabolites were identified by comparing the retention time and the mass spectrum with those of commercially available reference standards (Sigma-Aldrich s.r.l., Milan, Italy) as well as with those homemade (Department of Chemical and Geological Sciences, University of Cagliari, Italy).

The MetaboAnalyst tool was used for the validation of the following HCMV models: controls *vs.* transmitters, controls *vs.* non-transmitters, transmitters *vs.* non-transmitters, and ACI (Asymptomatic Congenital Infection) *vs.* SCI (Symptomatic Congenital Infection).

#### 2.1.1 Controls *vs.* transmitters model

Estimation was performed using a pre-processing step consisting in the exclusion of features with missing data greater than 50%, and the evaluation of missing values lower than 50% as half of the minimum value measured. In order to identify and remove variables that are unlikely to be of use in data modeling, a filtering process was applied on data [7]. Relative standard deviation (RSD=SD/mean) was applied to identify uninformative variables in dataset. Successively, data were normalized with a 3 steps procedure: i) samples normalization to the sum of all the acquired values as a general-purpose adjustment for the differences among samples; ii) data transformation through a generalized logarithm transformation; iii) scaling procedure by means of auto scaling procedure. A PLS-DA model was generated; healthy subjects were labeled as class 1, and transmitters as class 2. The maximum number of components calculated for the classification was 5. The corresponding R2, and Q2 values are reported below for each component (Table 1).

**Fig. 1:**
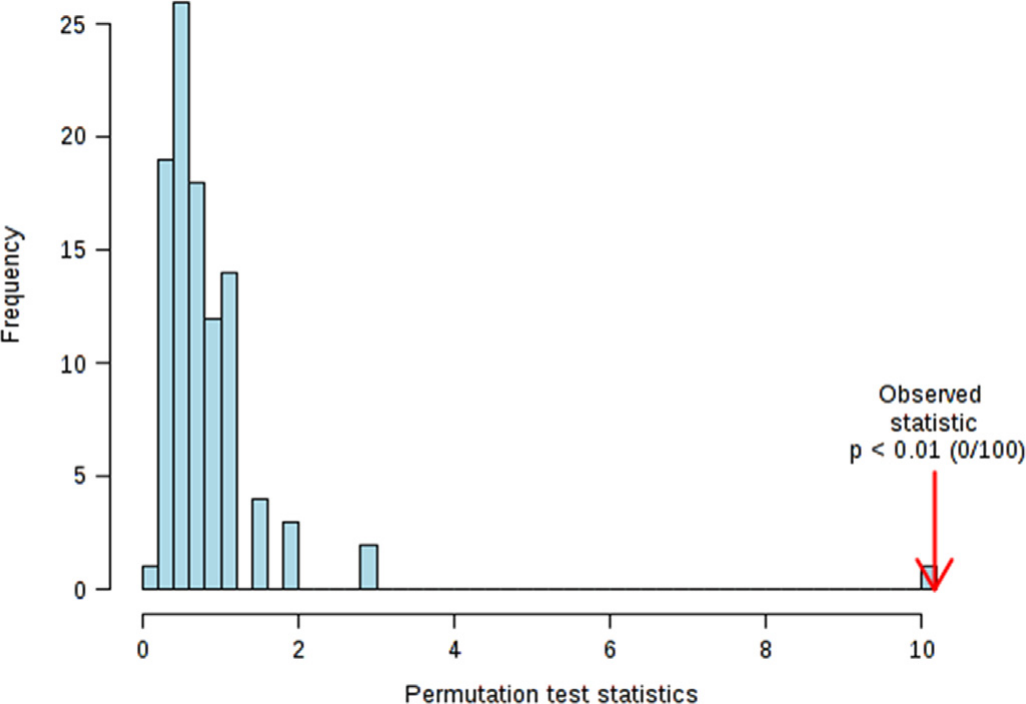
Permutation test; Select test statistic: Separation distance (B/W), set permutation numbers:100 *p*<0.01.

The Cross validation (CV) method employed for this study was the 10-fold CV, with Q2 as measured performance. Since PLS-DA tends to over fit data, the model needs to be validated in order to understand whether the separation is statistically significant or it is due to random noise. This hypothesis was tested using the permutation tests: in each permutation, a PLS-DA model is built between the data (*X*) and the permuted class labels (*Y*) using the optimal number of components determined by previous cross validation calculations and based on the original class assignment. The ratio of the between sum of the squares and the within sum of squares (B/W-ratio) is calculated for the class assignment prediction of each model. If the B/W ratio of the original class assignment is a part of the distribution based on the permuted class assignment, the contrast between the two class assignments cannot be considered significant from a statistical point of view. The following graph, suggested by Bijlsma et al. [8], helps to evaluate whether a class assignment is appropriate or misplaced. The histogram in Fig. 1 shows the distribution derived from the permuted samples. The highlighted bar represents the original sample. The further to the right of the distribution, the more significant is the separation between the two groups.

**Fig. 2:**
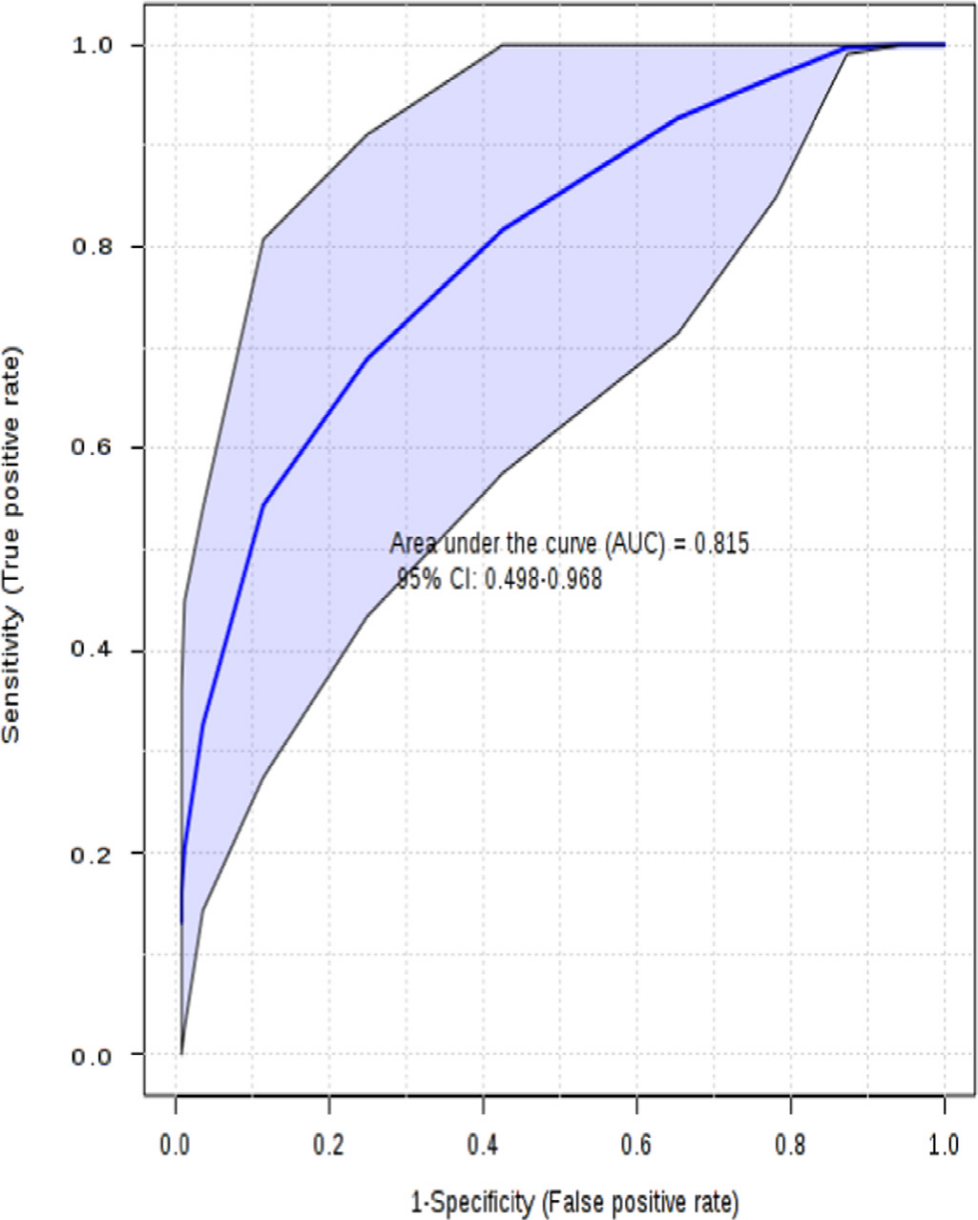
ROC curves, based on the cross validation (CV) performance. The ROC curve is the curve for the model with the least number of features (2), with 95% confidence interval computed for the model.

By using the Biomarker Analysis tool, it is possible to develop the ROC analysis for the model (Fig. 2). Multivariate ROC plot based exploratory analysis (Explorer) performs automated important feature identification, and performance evaluation. ROC curve analyses are based on partial least squares discriminant analysis (PLS-DA). ROC plots are generated by Monte-Carlo cross validation (MCCV) using balanced sub-sampling. In each MCCV, two thirds (2/3) of the samples are employed to evaluate the feature importance. The top 2, 3, 5, 10 …100 (max) important features are then exploited to build classification models, which are validated on the remaining 1/3 of the samples. The procedure is replicated many and many times in order to calculate performance, and confidence interval of each model. Several algorithms are available for classification, and feature ranking methods: in our calculations, the feature ranking method, PLS-DA algorithm with two latent variables (LV) was applied.

#### 2.1.2 Controls *vs.* non-transmitters model

The methods used in this data set are the same used in the previous model. The 10-fold Cross validation, with Q2 as measured performance, was again the CV method of choice (Table 2).

**Fig. 3:**
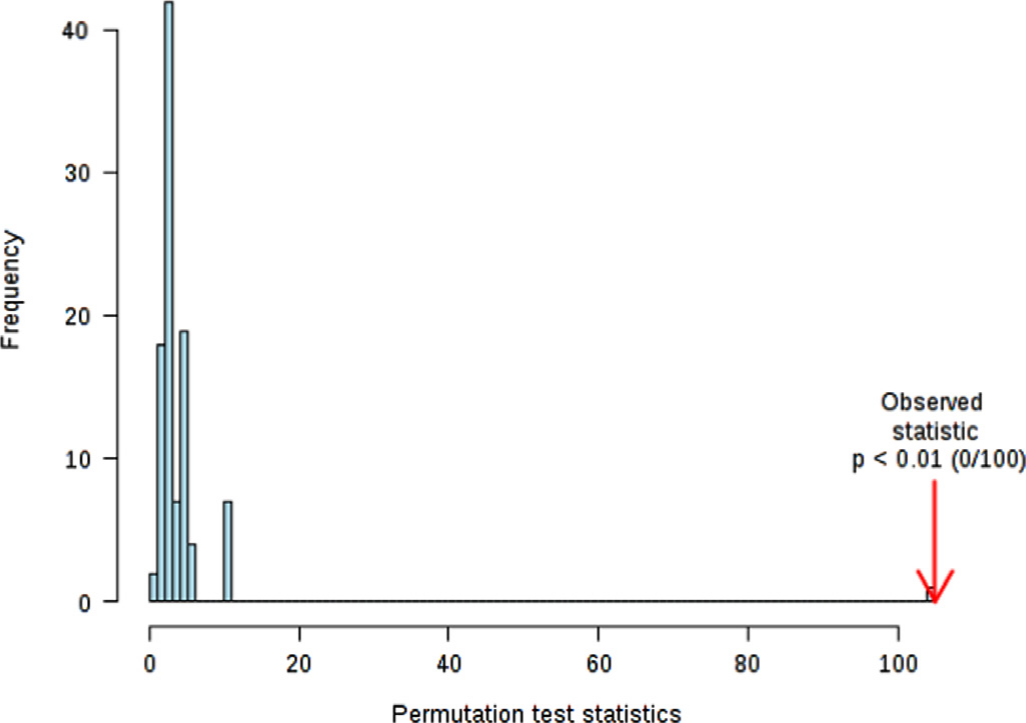
Permutation Test. Select test statistic: Separation distance (B/W), set permutation numbers:100 *p*<0.01.

**Fig. 4:**
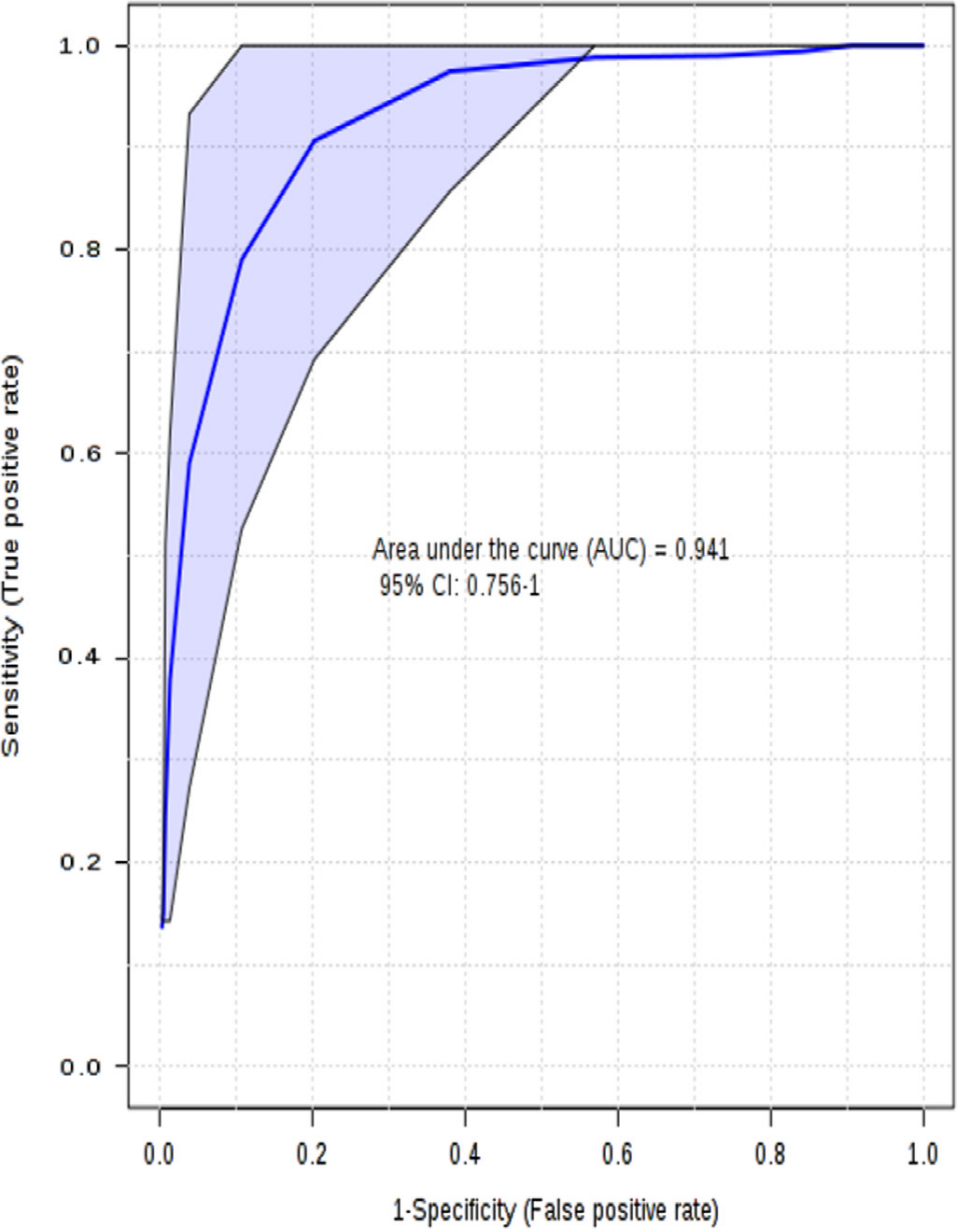
ROC plot for the PLS-DA model.

The permutation test is showed in Fig. 3:

The histogram in Fig. 3 shows the distribution of the permuted samples. In this case, the highlighted bar is close to the right side of the distribution, meaning a statistically significant separation between the two groups. Also the ROC analysis for the model with 2 features delivers good values, with an area under the curve (AUC) equal to 0.94 (Fig. 4).

#### 2.1.3 Transmitters *vs.* non-transmitters model

The whole set of methods applied for these data is the same of that used for the previous models (Table 3).

Since this model shows unusual Q2 residuals; such values should be investigated. In the case it is not possible, the advice is either to use all the latent variables available or to add more samples for a more reliable model.

**Fig. 5:**
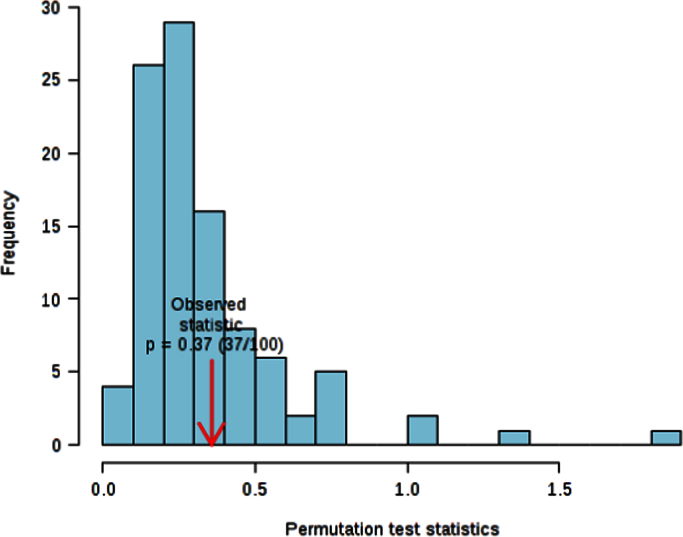
Permutation test.

The histogram in Fig. 5 shows the distribution of the permuted samples. In this case, the highlighted bar is close to the left side of the distribution and this means a statistically non-significant separation between the two groups (*p*=0.37). Also the ROC curve analysis for the model with 2 features gave an unsatisfactory result.

#### 2.1.4 ACI *vs.* SCI mode

**Fig. 6:**
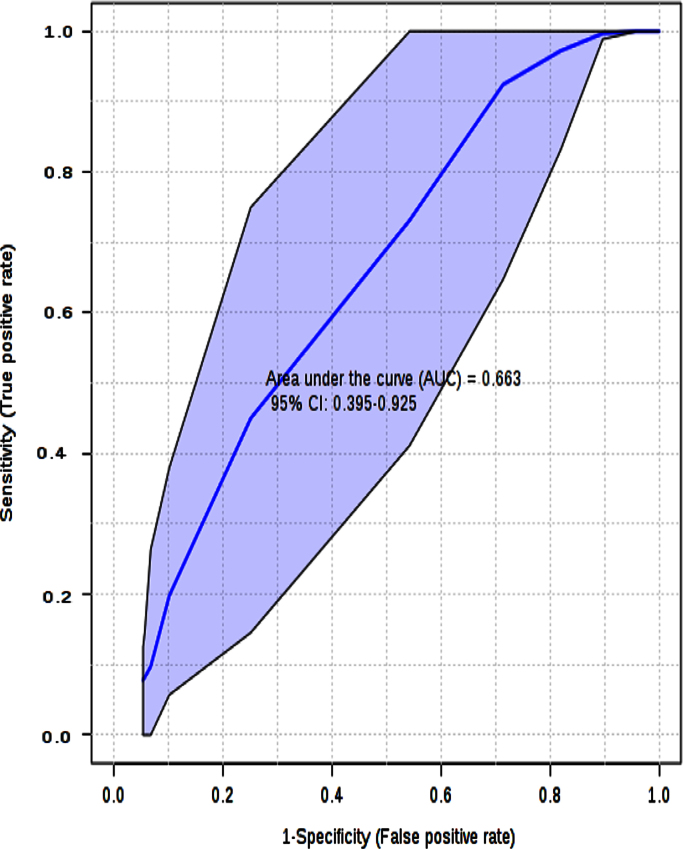
ROC plot for the model (AUC=0.663).

The same statistical methods chosen for the previous models were applied to these data (Fig. 6).

PLS-DA cross validation for the comparison between ACI *vs.* SCI is reported in Table 4.

Similarly to the previous model, unusual Q2 residuals are observed. Again, investigation on Q2 residuals, and the employment of scores plot on “alien samples”/outliers may help to remove these “errors”. If this is not possible, the addition of other samples or the inclusion of much more latent variables (principal components) should be considered for a more reliable model.

**Fig. 7:**
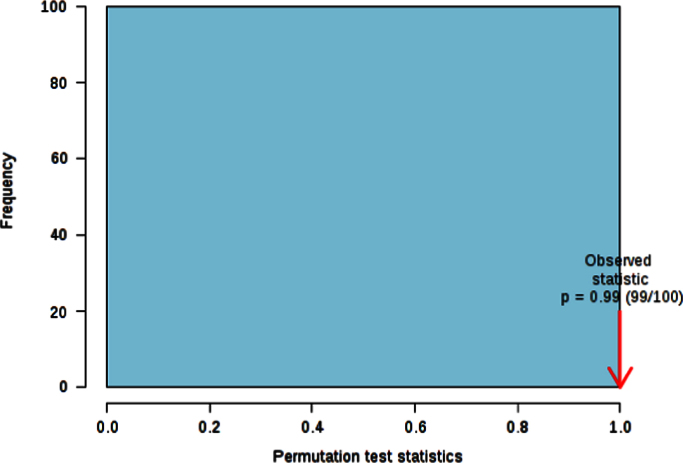
Permutation test.

**Fig. 8:**
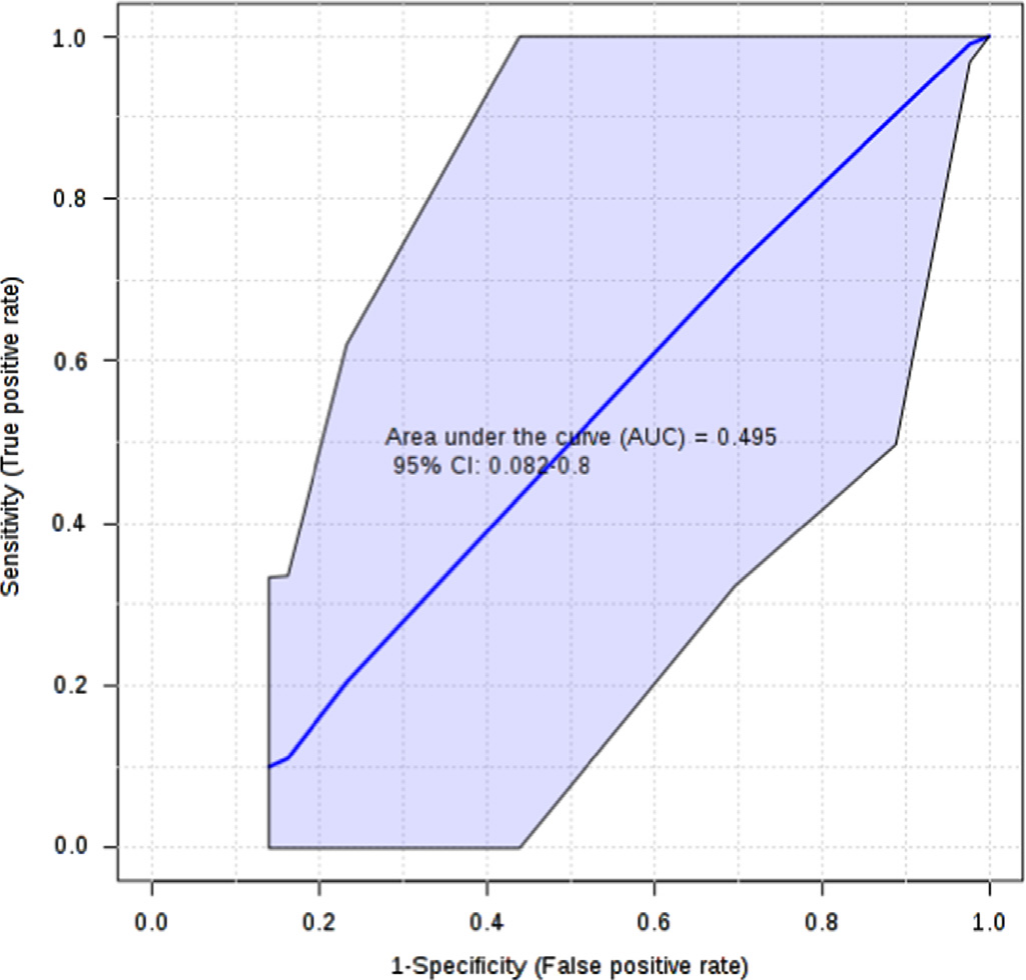
ROC plot for the model (AUC=0.495).

The histogram in Fig. 7 shows the distribution of the permuted samples. In this case, the highlighted bar is close to the left side of the distribution, representing a statistically non-significant separation between the two groups (*p*=0.99). Furthermore, the ROC curve calculations for the model with 2 features delivered an unsatisfactory value (Fig. 8).

**Fig. 9:**
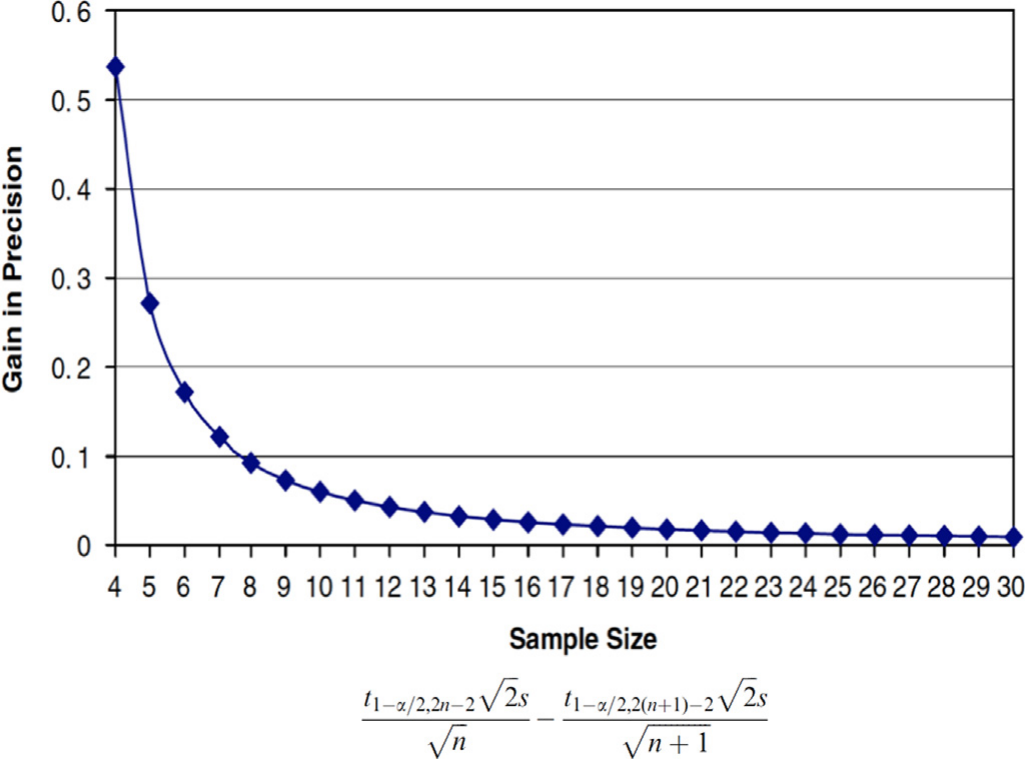
Increase of the precision for each unit in the sample size per group.

The problems reported in this case are probably related to the low number of classes in comparison with the intensity of the perturbation to be measured. Basically, a preliminary pilot study for revealing the presence of the perturbation of interest is recommended. The calculation of the pilot study size samples is performed considering the “mean precision-based samples size evaluation” [9]. The increase of the precision for each unit in the sample size per group is described by the algorithm and the rules in Fig. 9.

According to this approach, the minimum sample size selected in an exploratory trial for the classes is 12 (no prior information). The rationale is based on the precision requested for the groups mean. Our calculation suggests that the perturbation of interest may be satisfactorily described by our sample size in the firsts two models. However, for the differences between transmitter and non-transmitters, and ACI *vs.* SCI the precision is not suitable to reveal the perturbation of interest with statistical significance. For these reasons, Power Analysis were performed for each of the four models, using the proper tool in MetaboAnalyst. Given that a certain effect is present, the Power may be defined as the probability of detecting that particular effect. For instance, if a study comparing two groups (healthy *vs.* diseased) has a power of 0.8, assuming that the experiments may be conducted several times, a statistically significant difference between the two groups should be detected 80% of the time. Therefore, despite the presence of that effect, 20% of the experiments should not highlight a statistically significant effect. There are three major factors affecting the Power Analysis:

(a) effect size, which is usually defined as the difference of two group means divided by the pooled standard deviation. When the other factors are equivalent in the groups under study, a larger effect size will lead to more power.
(b) degree of confidence, which is usually the *p*-value cut-off (alpha) for statistical significance. When the other factors are equivalent in the groups under study, reduced power will be observed when a very high degree of confidence is required.
(c) sample size. Typically, a larger number of samples increases the power. In several cases, the sample size is of interest for a given power (i.e. 0.8).

**Fig. 10:**
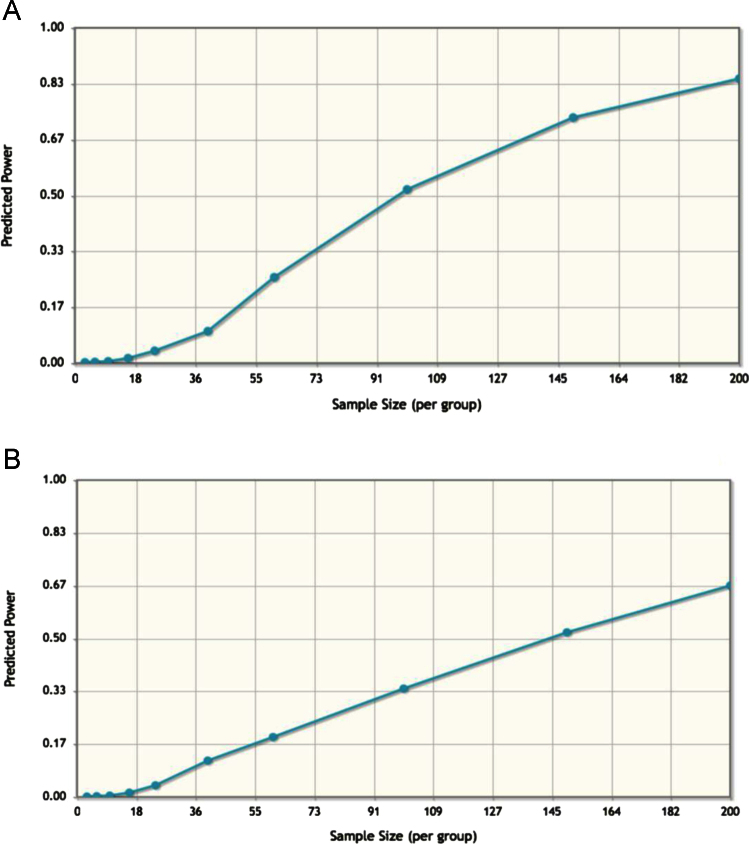
Estimation of the effect size in the models. A= transmitters *vs.* non-transmitters. B= ACI *vs.* SCI.

Taking into account the comparison between transmitters *vs.* non-transmitters, and ACI *vs.* SCI, with a sample size of 200 subjects, we computed a predicted power of 0.84 for the former model and 0.67 for the latter. Therefore the number of samples should be further increased (Figs. 10a, b).

The effect size is estimated for the “pilot” study [1]. The graph allows for the investigation of the sample size *vs.* the statistic power, guiding the study design. The algorithm allows for the exploration of a range of sample sizes and, following specification of FDR, a graph is produced in order to show the power of analysis in relation to the sample size applied.

## Figures and Tables

**Table 1:** R2 and Q2 values for controls *vs.* transmitters model.

**Measure**	**1 comps**	**2 comps**	**3 comps**	**4 comps**	**5 comps**
**Accuracy**	0.84	0.88	0.86	0.9	0.88
**R2**	0.59105	0.75522	0.84065	0.88317	0.9086
**Q2**	0.43483	0.58123	0.46706	0.37141	0.28953

**Table 2:** R2 and Q2 values for controls *vs.* non-transmitters model.

**Measure**	**1 comps**	**2 comps**	**3 comps**	**4 comps**	**5 comps**
**Accuracy**	0.86	0.88	0.88	0.92	0.88
**R2**	0.70368	0.78236	0.87182	0.91832	0.94438
**Q2**	0.49923	0.54274	0.57553	0.62088	0.6229

**Table 3:** R2 and Q2 values for transmitters *vs.* non-transmitters model.

**Measure**	**1 comps**	**2 comps**	**3 comps**	**4 comps**	**5 comps**
**Accuracy**	0.46	0.44	0.46	0.46	0.44
**R2**	0.35779	0.46634	0.58919	0.69187	0.79734
**Q2**	−0.10805	−0.13862	−0.36437	−0.63298	−1.0741

**Table 4:** R2 and Q2 values for ACI *vs.* SCI model.

**Measure**	**1 comps**	**2 comps**	**3 comps**	**4 comps**	**5 comps**
**Accuracy**	0.5	0.45	0.5	0.65	0.6
**R2**	0.35271	0.75519	0.91092	0.96485	0.98726
**Q2**	−0.15551	−0.33554	−0.25473	−0.098897	−0.04234

## Footnotes

Appendix A Supplementary data associated with this article can be found in the online version at http://dx.doi.org/10.1016/j.dib.2016.08.050.
