# Supplementary material for: Multivariate data validation for investigating primary HCMV infection in pregnancy
Source: Data Brief. 2016 Aug 31;9:220–30. doi: 10.1016/j.dib.2016.08.050 (PMC5021794; doi:10.1016/j.dib.2016.08.050)
Supplement: Supplementary file 1 — Supplementary material [file mmc1.doc]

**CONFLICT OF INTEREST**

Authors declare the absence of any potential conflicts of interest, including employment, consultancies, stock ownership, honoraria, paid expert testimony, patent applications/ registrations, and grants or other funding.

Briefly: Conflicts of interest: none
